# Emergency Department Operational Measures Associated with EMS Redirection Activation and Duration

**DOI:** 10.5811/westjem.20766

**Published:** 2026-10-14

**Authors:** Barry Hahn, Anna Van Tuyl, Josh Greenstein, Kurien Mathews, Arsalan Shawl, Paul Barbara, Amin Mohamadi

**Affiliations:** * Northwell Health, New Hyde Park, New York; † Staten Island University Hospital, Northwell Health, Department of Emergency Medicine, Staten Island, New York; ‡ Hadassah University Medical Center - Ein Kerem, Department of Emergency Medicine, Jerusalem, Israel

## Abstract

**Introduction:**

Emergency medical services (EMS) redirection is used to manage emergency department (ED) strain, but whether it reflects operational conditions is unclear. We compared associations of prespecified ED operational measures, including visit volume, patient flow times, admissions, boarding, and left-early measures, with redirection activation and total daily redirection minutes.

**Methods:**

We performed a retrospective study at a single urban tertiary care center over 392 consecutive days from April 7, 2021 through May 3, 2022. Redirection temporarily routed ambulances away from the study hospital and was activated centrally by EMS. New York City’s 9-1-1 system included about 500 ambulance units and over 60 hospitals. The primary outcome was activation, defined as any redirection during a calendar day. The secondary outcome was total redirection minutes among days with redirection. Multivariable logistic regression evaluated activation, and negative binomial regression evaluated duration.

**Results:**

Redirection occurred on 250 of 392 days (63.8%). Median daily duration was 47 minutes (interquartile range 23–78). Higher ED visit volume was associated with activation (odds ratio [OR] 1.14 per 10 patients; 95% CI, 1.02–1.27). Longer door-to-room time was associated with activation (OR 1.42 per 5 min; 95% CI, 1.04–1.92) and duration (incidence rate ratio 1.022 per min; 95% CI, 1.002–1.042). Boarding time and admission count were not associated with either outcome.

**Conclusion:**

Emergency department visit volume was associated with activation but not duration, while door-to-room time was associated with both. Evaluating redirection as a single outcome may obscure differences between activation and cumulative duration.

## INTRODUCTION

Emergency medical services (EMS) diversion and redirection alter ambulance destination in response to operational strain. In this study, redirection refers to the temporary rerouting of ambulances away from the study hospital to other receiving hospitals. Prior studies have associated ambulance diversion with delays in care and worse outcomes in acute myocardial infarction.^1^,^2^ Reviews have found mixed evidence regarding broader EMS and patient outcomes and limited guidance on how diversion should be operationalized.^3^,^4^

Ambulance diversion policies vary across EMS systems. In the more commonly described model, individual hospitals request diversion when emergency department (ED) or hospital capacity is limited, although regional EMS authorities may coordinate, restrict, or override these requests. Centralized destination-management programs have also been described.^4^–^6^ Fully centralized systems in which EMS activates and discontinues redirection without hospital input are less frequently described in the literature. However, the relative prevalence of these governance models has not been systematically quantified.

In New York City, the Fire Department of New York (FDNY) operates a centralized EMS system with a coordinated redirection process that differs from traditional hospital-initiated diversion. Individual EDs do not initiate or discontinue redirection. Redirection is activated when EMS operational criteria related to ambulance availability and hospital turnaround are met and remains active until those conditions resolve. While redirection is active, ambulances initiating patient transport generally bypass the affected hospital and are directed to another receiving facility.^7^ The New York City 9-1-1 EMS system includes approximately 500 ambulance units and more than 60 hospitals in its network.^8^

Most studies of ED crowding and ambulance diversion have evaluated ambulance destination restriction as a single outcome and examined its association with measures of input, throughput, and output, including patient volume, length of stay (LOS), and boarding.^3^,^9^ However, EMS redirection may represent two distinct processes: activation and duration. Activation indicates whether redirection is initiated, while duration reflects the total time spent on redirection once it is initiated. Activation and duration likely reflect different aspects of system function, with activation more closely aligned with system-level demand and duration more closely aligned with ED throughput. Prior studies have not clearly examined these components separately.

Separating operational measures associated with redirection activation from those associated with redirection duration may clarify whether current redirection practices align with ED conditions and may help refine activation criteria. Evaluating redirection as a single outcome may conceal differences between activation and cumulative duration and limit efforts to improve system performance. In this study, we compared prespecified ED operational measures associated with EMS redirection activation and total daily redirection minutes at a single urban tertiary care center. We hypothesized that the operational measures associated with activation would differ from those associated with duration, reflecting distinct processes underlying redirection activation and cumulative duration.

## METHODS

### Study Design and Setting

We performed a retrospective observational study of EMS redirection and ED operational metrics at a single urban tertiary care hospital in New York City. This was a single-site study and did not evaluate redirection across the broader EMS system. The study period extended from April 7, 2021, through May 3, 2022, and included all consecutive calendar days (*N* = 392). The hospital is an academic center with approximately 97,000 annual ED visits and is one of three 9-1-1 receiving hospitals in the borough. The institutional review board approved the study with a waiver of informed consent under the original study protocol (Protocol# 23-0329). This secondary analysis was conducted under the same protocol.

**Population Health Research Capsule**

What do we already know about this issue?

*Ambulance diversion is used to manage crowding, but the emergency department (ED) operational conditions associated with redirection activation and duration are unclear.*

What was the research question?

*Which ED operational measures are associated with emergency medical services (EMS) redirection activation versus duration?*

What was the major finding of the study?

*Each 5-minute longer door-to-room time increased activation odds: OR 1.42 (95% CI, 1.04–1.92), P = .03.*

How does this improve population health?

*Identifying ED flow measures aligned with EMS redirection may help systems recognize strain and improve ambulance destination and flow decisions.*

### Data Sources

We obtained EMS redirection status and timestamps from automated notifications generated by the FDNY. Redirection was determined centrally by the FDNY and was not initiated by the ED. Under the threshold assigned to the study hospital during the study period, redirection was activated when units from three incidents had remained at the hospital for at least 30 minutes and was discontinued when units from no more than one incident remained beyond the 30-minute turnaround threshold.^7^ The FDNY threshold reflected the time that an ambulance unit remained at the hospital rather than a direct measure of the interval from ambulance arrival to transfer of patient care.^10^ The daily hospital operational measures evaluated in this study, including visit volume, admission count, LOS measures, boarding time, door-to-triage time, door-to-room time, and left-early measures, were not explicit components of the formal FDNY activation or discontinuation rule.

All study variables were obtained from existing electronic data sources. A study investigator exported EMS redirection status and timestamps from the FDNY automated email notification log into a structured dataset. Emergency department operational measures were extracted from the institutional emergency medicine service line data repository by the departmental analytics team using standardized queries. Thus, the two data sources were extracted independently by different personnel and subsequently merged by calendar date. For each study day, we calculated total redirection duration by summing all intervals between activation and discontinuation. Redirection activation and discontinuation timestamps were available at the episode level. For the primary analysis, episode-level redirection intervals were aggregated to derive daily redirection activation and total daily redirection minutes. Emergency department operational measures were available only as daily summary values. No manual chart abstraction or patient-level research extraction was performed.

The analytic dataset was reviewed for duplicate dates, missing values, and values outside expected ranges. To verify extraction accuracy, a simple random sample of 20 study days, representing 5% of the study period, was independently compared by a study investigator with the corresponding FDNY notification logs and institutional operational reports. No discrepancies were identified. All 392 consecutive study days were represented, and no ED operational measures included in the analyses had missing values. Redirection duration was not defined on days without redirection because duration was analyzed only among redirection days. No imputation was performed. Although selected aggregate values were verified, the underlying operational systems were not independently validated for this study.

This analysis used the same underlying dataset as a prior study examining associations between ED operational measures and EMS redirection.^7^ The present study addresses a distinct question by evaluating redirection activation separately and comparing the operational measures associated with activation and duration.

### Outcomes

We evaluated two related outcomes. The first was redirection activation, defined as any EMS redirection during a calendar day. The second was redirection duration, defined as the total number of redirection minutes per day. Activation was evaluated across all study days, whereas duration was evaluated among days with redirection.

### Emergency Department Operational Measures

We prespecified ED operational measures based on operational relevance and prior literature on ED crowding.^9^ Daily ED visit volume, admission count, and counts of patients who left without being seen, left against medical advice, or eloped were obtained from the institutional emergency medicine service line data repository. Total ED LOS, discharge LOS, boarding time, door-to-triage time, and door-to-room time were obtained from the same repository as daily average values in minutes.

We defined total ED LOS as the interval from ED registration to departure for all encounters. Discharge LOS was defined as the interval from ED registration to departure among encounters with a final disposition of discharge. Boarding time was defined as the interval from the decision to admit until physical departure from the ED to an inpatient location. We defined door-to-triage time as the interval from registration to completion of triage. Door-to-room time was defined as the interval from completion of triage to placement in an emergency department treatment location. We. calculated each time-based measure as a daily average.

A composite left-early measure was calculated as the sum of patients who left without being seen, left against medical advice, or eloped during each calendar day.

### Statistical Analysis

We summarized continuous variables using medians and interquartile ranges (IQR) or means and standard deviations (SD), as appropriate. Categorical variables were summarized using frequencies and percentages. We compared operational measures between days with and without redirection using absolute standardized mean differences. Among days with redirection, unadjusted associations between operational measures and redirection duration were assessed using Spearman rank correlation coefficients. We evaluated associations between the prespecified operational measures and redirection activation and duration separately. To evaluate associations with redirection activation, we used multivariable logistic regression across all study days. Results are reported as odds ratios (OR) with 95% CIs.

To evaluate associations with redirection duration, we used negative binomial regression among days with redirection. This model was selected because the outcome demonstrated overdispersion, as indicated by comparisons of the variance to the mean and model fit diagnostics. Results are reported as incidence rate ratios (IRR) with 95% CIs. The activation model included daily ED visit volume, admission count, boarding time, door-to-room time, and day of week. The duration model included daily ED visit volume, admission count, discharge LOS, boarding time, door-to-room time, and day of week.

Covariates included in the multivariable models were selected a priori based on clinical and operational relevance. We limited the number of variables included in multivariable models to maintain an appropriate ratio of observations to model covariates. Correlation among covariates was assessed using variance inflation factors. When collinearity was present, defined as a variance inflation factor greater than 5, one variable from the correlated pair was excluded based on interpretability and prior evidence. For example, total ED LOS and boarding time were highly correlated, and only boarding time was retained in multivariable models.

We performed a prespecified sensitivity analysis excluding days with door-to-room time in the top 5% to assess the influence of extreme values. Results were compared with the primary model to assess robustness.

In a post hoc sensitivity analysis, we calculated the average redirection episode duration for each redirection day by dividing total daily redirection minutes by the number of redirection activations. We modeled average episode duration using generalized linear regression with a gamma distribution and log link, adjusting for the same covariates as the primary duration model. Robust standard errors were used.

In additional post hoc sensitivity analyses, we repeated the activation, total-duration, and average-episode-duration models with continuous calendar time, scaled per 100 study days, included as an additional covariate to assess potential secular trends during the study period.

Model fit was assessed using standard goodness-of-fit diagnostics. All tests were two-sided, and *P* values < .05 were considered statistically significant. We performed analyses using StataNow SE, version 19.5 (StataCorp). This study is reported in accordance with the Strengthening the Reporting of Observational Studies in Epidemiology guidelines.

## RESULTS

The study period included 392 calendar days. Emergency medical services redirection occurred on 250 days (63.8%), while 142 days (36.2%) had no redirection. Among days with redirection, the median daily redirection duration was 47 minutes (IQR 23–78 minutes), and the mean duration was 58.2 minutes (SD 44.6 minutes). There were 433 redirection activations and 14,544 total redirection minutes (242.4 hours) during the study period. The median number of activations per redirection day was 1 (IQR 1–2; range 1–5), and 116 of 250 redirection days (46.4%) had more than one activation.

### Comparison of Days with and Without Redirection

Descriptive characteristics by redirection status are shown in Table 1. Days with redirection had a higher median daily ED visit volume, longer door-to-room time, and longer door-to-triage time than days without redirection. Median daily ED visit volume was 245 (IQR 224–265) on redirection days versus 227 (IQR 208–242) on no-redirection days. Median door-to-room time was 18 minutes (IQR 15–22 minutes) versus 15 minutes (IQR 14–17 minutes), and boarding time was 510 minutes (IQR 379–647 minutes) versus 475 minutes (IQR 343–631 minutes). Admission count, total ED LOS, discharge length of stay, and left-early measures were higher on redirection days. Absolute standardized mean differences are presented in Table 1.

### Operational Measures Associated with Redirection Activation

Multivariable logistic regression results for redirection activation (*N* = 392 days) are shown in Table 2. Higher daily ED visit volume and longer door-to-room time were independently associated with redirection activation. For every 10-patient increase in daily ED visit volume, the odds of redirection activation were higher (OR 1.14; 95% CI, 1.02–1.27; *P* = .02). For every 5-minute increase in door-to-room time, the odds of redirection activation were higher (OR 1.42; 95% CI, 1.04–1.92; *P* = .03). Boarding time, admission count, and day of the week were not independently associated with redirection activation.

### Operational Measures Associated with Redirection Duration

Unadjusted associations with redirection duration are presented in Supplemental Table 1. In multivariable negative binomial regression among redirection days (*n* = 250), door-to-room time was independently associated with duration (Table 3). Each 1-minute increase in door-to-room time was associated with a 2.2% increase in duration (IRR 1.022; 95% CI, 1.002–1.042; *P* = .04). Daily ED visit volume, admission count, discharge LO0S, boarding time, and day of week were not independently associated with duration. In a sensitivity analysis excluding days in the highest 5% of door-to-room time, effect estimates and statistical significance were unchanged. In a post hoc sensitivity analysis evaluating average redirection episode duration, door-to-room time remained independently associated with longer episodes (ratio 1.020 per minute; 95% CI, 1.005–1.036; *P* = .009). The principal associations were also unchanged after adjustment for continuous calendar time. Emergency department visit volume and door-to-room time remained associated with redirection activation, and door-to-room time remained associated with both total daily redirection duration and average episode duration. Calendar time was not independently associated with any of the three outcomes.

## DISCUSSION

In this single-center analysis of 392 calendar days, EMS redirection occurred on most days. It was more likely on days with higher ED visit volume and longer door-to-room time. Door-to-room time was the only variable independently associated with both the occurrence and duration of redirection. Daily ED visit volume was associated with redirection activation but not with redirection duration. Other operational measures, including boarding time and admission count, were not independently associated with either outcome after adjustment. These findings suggest that door-to-room time is associated with both the activation and duration of EMS redirection.

Emergency medical services redirection is often described as a response to ED crowding, but the specific operational signals associated with its use are not well defined. Evaluating redirection as a single outcome may obscure differences between activation and cumulative duration, which appear to reflect distinct operational processes. Door-to-room time likely reflects multiple overlapping operational factors, including triage throughput, room availability, staffing, and patient intake flow, rather than a single modifiable factor. Its association with both activation and duration is consistent, with a role for early intake constraints in the conditions under which redirection occurs.

Redirection in this setting was triggered by EMS operational metrics rather than hospital request. The FDNY threshold was based on ambulance-unit turnaround and did not directly include the daily hospital operational measures examined. Door-to-room time nevertheless remained associated with redirection activation, total daily duration, and average episode duration. These findings suggest alignment between EMS-facing ambulance turnaround conditions and ED intake performance, but the findings do not establish that hospital measures caused FDNY redirection decisions. Most published diversion models involve hospital-initiated requests, sometimes coordinated or restricted by regional EMS authorities, while centralized destination-management programs have also been described.^3^–^6^ Because ambulance destination policies differ across EMS systems, the findings may not generalize directly to systems in which individual hospitals control diversion status.

Prior work has examined hospital contributors to ambulance diversion duration using shorter operational intervals. Schull et al found that boarded patients, admissions, assessment time, and boarding time were associated with diversion minutes measured during consecutive eight-hour intervals.^11^ In contrast, boarding time and admission count were not independently associated with redirection in our study, while door-to-room time was associated with both occurrence and duration. This pattern suggests that redirection was more closely aligned with early intake conditions than with downstream congestion or admission volume. These differences may reflect the centralized EMS-controlled decision rule, differences in temporal aggregation, or variation in the operational processes underlying hospital-initiated diversion and EMS-initiated redirection.

Prior studies have linked ED crowding and ambulance diversion to delays in care and adverse outcomes.^1^–^4^,^9^,^12^ A recent scoping review found that evidence regarding the effects of ambulance diversion on EMS and patient outcomes remains inconclusive and noted a lack of operational guidance for its use.^4^ In a prior analysis of the same dataset, daily ED visit volume had only a small association with redirection occurrence, while other operational measures were not significantly associated.^7^ The present analysis extends that work by evaluating activation and duration separately and identifying door-to-room time as the only measure associated with both outcomes.

Related studies suggest that the operational measures associated with restricting patient inflow depend on the type of decision being made. Greenwood-Ericksen et al found that greater ED boarding and inpatient census were associated with lower interhospital transfer acceptance.^13^ In contrast, boarding time was not independently associated with either redirection activation or duration in our study. This difference may reflect the influence of downstream hospital capacity on transfer acceptance, whereas ambulance turnaround conditions triggered FDNY redirection. Feral-Pierssens et al found that redirecting low-acuity ED patients to nearby clinics was associated with shorter time to initial assessment and fewer patients leaving without being seen, but not with improved overall ED LOS.^14^ Although this represents a different form of redirection, the findings similarly suggest that strategies affecting patient input or early flow do not necessarily resolve downstream congestion. In our study, redirection appeared more closely associated with early intake strain than with boarding or admission volume.

## LIMITATIONS

This was a single-site study conducted at one hospital within the New York City EMS system and did not evaluate systemwide redirection patterns. The centralized EMS-controlled process may limit external validity because ambulance destination policies vary across jurisdictions, and the findings may not generalize to systems in which individual hospitals initiate and discontinue diversion. The study period occurred during the COVID-19 pandemic, when ED utilization and case mix differed from prepandemic patterns. National ED volumes remained below 2019 levels through early 2022.^15^ Therefore, the magnitude and pattern of the observed associations may not reflect current operational conditions.

The study used day-level aggregation and did not include patient-level outcomes or direct measures of EMS performance. Although redirection timestamps were available at the episode level, ED operational measures were available only as daily summary values. The analysis therefore could not establish temporal ordering or assess within-day variation. Short periods of severe crowding or prolonged door-to-room time may have been diluted by lower values during other portions of the day, potentially attenuating associations with redirection episodes. Selected aggregate values were verified against source records; however, the underlying operational systems were not independently validated, and undetected source-system or aggregation errors remain possible.

Although redirection was frequent, the median total daily duration on redirection days was 47 minutes. The study was not designed to determine whether redirection totaling this duration affected ED function, EMS performance, or patient outcomes. Neither did the analysis include patient acuity, staffing levels, or real-time ED capacity. Door-to-room time likely represents a composite operational signal rather than a direct mechanistic driver of redirection. Future multicenter studies using time-matched EMS and hospital data should evaluate whether interventions targeting door-to-room time affect redirection, EMS performance, or patient outcomes.

## CONCLUSION

In this single-center analysis, EMS redirection was most consistently associated with door-to-room time, which was independently associated with both activation and duration. In contrast, emergency department visit volume was associated with activation but not duration. Evaluating redirection as a single outcome may obscure differences between activation and cumulative duration. Door-to-room time may be a practical indicator of operational strain relevant to both hospital flow and EMS coordination.

## Supplementary Information



## Figures and Tables

**Table 1:** Emergency department operational characteristics by emergency medical services (EMS) redirection status (*N* = 392 calendar days) in a study of EMS redirection activation versus duration at a single urban emergency department.

Variable	No redirection days (*n* = 142), median (IQR)	Redirection Days (*n* = 250), median (IQR)	Absolute SMD	Missing *n* (%)
Daily ED volume	227 (208–242)	245 (224–265)	0.656	0 (0.0)
Admission count	53 (48–59)	59 (53–65)	0.513	0 (0.0)
LWBS	1 (0–2)	1 (0–3)	0.252	0 (0.0)
AMA	3 (2–4)	3 (2–5)	0.148	0 (0.0)
Eloped	3 (2–4)	3 (2–5)	0.225	0 (0.0)
Left early	8 (5–9)	9 (6–11)	0.331	0 (0.0)
Total ED LOS, minutes	380 (340–417)	396 (356–439)	0.333	0 (0.0)
Discharge LOS, minutes	224 (209–240)	228 (214–248)	0.308	0 (0.0)
Boarding time, minutes	475 (343–631)	510 (379–647)	0.194	0 (0.0)
Door-to-room time, minutes	15 (14–17)	18 (15–22)	0.595	0 (0.0)
Door-to-triage time, minutes	7 (6–8)	8 (6–9)	0.380	0 (0.0)

Left early = sum of patients who left without being seen, left against medical advice, or eloped.

*AMA*, against medical advice; *ED*, emergency department; *IQR*, interquartile range; *LOS*, length of stay; *LWBS*, left without being seen; *SMD*, standardized mean difference.

**Table 2:** Multivariable logistic regression analysis of operational measures associated with emergency medical services (EMS) redirection activation (*N* = 392 days) in a study of EMS redirection activation versus duration at a single urban emergency department.

Variable	OR	Lower 95% CI	Upper 95% CI	*P* value
Daily ED volume (per 10 patients)	1.14	1.02	1.27	.02
Door-to-room time (per 5 min)	1.42	1.04	1.92	.03
Boarding time (per 100 min)	0.96	0.85	1.09	.56
Admission count (per 10)	1.12	0.81	1.54	.48
Friday vs Saturday	1.65	0.73	3.73	.23
Monday vs Saturday	1.58	0.59	4.21	.36
Sunday vs Saturday	1.58	0.70	3.53	.27
Thursday vs Saturday	1.90	0.81	4.43	.14
Tuesday vs Saturday	1.71	0.73	4.04	.22
Wednesday vs Saturday	1.71	0.71	4.14	.23

*ED*, emergency department; *OR*, odds ratio.

**Table 3:** Multivariable negative binomial regression analysis of operational measures associated with emergency medical services (EMS) redirection duration among days with redirection (*n* = 250 days) in a study of EMS redirection activation versus duration at a single urban emergency department.

Variable	IRR	Lower 95% CI	Upper 95% CI	*P* value
Sunday vs Saturday	0.811	0.556	1.183	.28
Monday vs Saturday	1.349	0.920	1.977	.13
Tuesday vs Saturday	1.169	0.823	1.661	.38
Wednesday vs Saturday	1.326	0.931	1.889	.12
Thursday vs Saturday	1.108	0.782	1.569	.56
Friday vs Saturday	1.414	0.992	2.015	.06
Daily ED volume, per 10 patients	1.033	0.996	1.072	.08
Admission count, per patient	0.996	0.985	1.007	.49
Discharge LOS, per hour	1.039	0.793	1.361	.78
Boarding time, per hour	1.024	0.996	1.052	.09
Door-to-room time (per 1 minute)	1.022	1.002	1.042	.04

IRR represents the relative change in redirection duration per unit increase.

Saturday was the reference day for day-of-week comparisons.

*ED*, emergency department; *IRR*, incidence rate ratio; *LOS*, length of stay.
